# A Comparative Study on 5hmC Targeting Regulation of Neurons in AD Mice by Several Natural Compounds

**DOI:** 10.1155/2020/5016706

**Published:** 2020-08-05

**Authors:** Dongyi Cao, Dewei Jiang, Dongming Zhou, Hong Yu, Jiali Li

**Affiliations:** ^1^Yunnan Herbal Laboratory, School of Ecology and Environmental Science, Yunnan University, Kunming, 650091 Yunnan, China; ^2^The International Joint Research Center for Sustainable Utilization of Cordyceps Bioresources in Southeast Asia, Yunnan University, Kunming Yunnan 650091, China; ^3^Key Laboratory of Animal Models and Human Disease Mechanisms of Chinese Academy of Sciences & Yunnan Province, Kunming Institute of Zoology, Chinese Academy of Sciences, Kunming, Yunnan 650223, China; ^4^Kunming Primate Research Center of the Chinese Academy of Sciences, Kunming, Yunnan 650223, China; ^5^Environment and Health Research Centre, Southwest China Eco-development Academy, Southwest Forestry University, Kunming, Yunnan 650224, China

## Abstract

A series of studies have confirmed that DNA methylation disorder (5mC/5hmC) is closely related to the occurrence and development of some diseases, such as Alzheimer's disease (AD). This study aims at discovering natural compounds that could adjust and control 5-hydroxymethylcytosine (5hmC) levels and improve Alzheimer's disease (AD) neuronal status. Cordycepin and cordycepic acid were selected as research materials, with resveratrol as positive control. The results of Dot Blot indicated that cordycepin, cordycepic acid, and resveratrol significantly increased the expression level of 5hmC. Combined with qPCR results, it was revealed that cordycepin increased the expression of ten-eleven translocation (TETs) mRNA compared with the abovementioned cordycepic acid and resveratrol. Besides, cordycepin dramatically reduced the transcription level of Apolipoprotein E (*ApoE*), suggesting that cordycepin might hinder the formation of NFTs (neurofibrillary tangles) and the accumulation of amyloid *β*-protein (A*β*) in the brain by reducing the expression of *ApoE*, resulting in affecting the progression of AD. Meanwhile, the immunofluorescence (IF) staining results demonstrated that the percentage of differentiation of SHSY-5Y cells reasonably increased after the treatment of cordycepin and cordycepic acid. Simultaneously, the length of axons and the number of dendritic branches in mouse primary neurons were substantially increased by cordycepin. The screening results illustrated that cordycepin had a positive influence on the level of 5hmC and the morphology of neurons, and most of the effects were better compared to the positive control (resveratrol). It indicated that cordycepin delayed the progression of neurodegenerative diseases such as AD. However, the specific mechanism of action still needs to be further investigated. Our research provided a foundation for further discussion about the influence of cordycepin on AD and a new idea for the pathological study of related diseases.

## 1. Introduction

AD is a neurodegenerative disease, which causes cognitive and memory deterioration, progressive impairment of activities of daily living. There are various pathological features of AD, such as neuronal loss and degeneration, amyloid plaques, and neurofibrillary tangles in the hippocampus and cortex [1]. World Alzheimer Report [2] illustrated that more than 9.9 million new cases of AD occurred in 2015, and the number will increase from 46 million to 131.5 million by 2050. The costs of AD in 2015 were around 818 billion dollars, AD became a trillion-dollar disease by 2018, and the cost will double by 2030. Most scholars believe that AD is likely to be caused by the interaction of various factors, including amyloid precursor protein (APP)/A*β*, *ApoE4*, tau, *α*-synuclein, tar DNA-binding protein 43, aging, and various complications. Unfortunately, the specific pathogenesis of Alzheimer's disease is still unclear. Therefore, coping with AD remains a huge challenge.

Nowadays, compounds for the treatment of AD are mainly chemical synthetic compounds or peptide compounds. Considering that there are toxic and side effects on the human body during long-term use and these compounds are often expensive, more and more attention has turned to natural compounds to explore more effective compounds for the treatment and prevention of AD.

*Cordyceps militaris* and *Cordyceps sinensis* are wildly used in traditional Chinese medicine; cordycepin and cordycepic acid were identified and proposed as significant active constituents [3]. Cordycepin, namely, 3′-deoxyadenosine, is a derivative of nucleotide and the main functional component of *Cordyceps militaris*. In recent years, studies have demonstrated that cordycepin has neuroprotective and neuromodulatory effects. Researchers [4] had revealed that oral cordycepin reduced the cerebral ischemia damage in CA1 and CA3 regions of hippocampal, and cordycepin was employed to treat neurodegenerative diseases by inhibiting the production of microglial inflammatory factors [5]. Cordycepic acid, also known as D-mannitol, is a natural compound from *Cordyceps sinensis*. There are several reports on the neuroprotective effect of mannitol on temporary or permanent vascular occlusion of cerebral ischemia. Current studies suggested that mannitol might reduce the ischemic neocortical injury and selective neuronal death in the forebrain and focal cerebral ischemia model [6]. Resveratrol was a polyphenolic compound mainly observed in grapes, peanuts, and mulberry plants; it had been verified to exhibit cardiovascular protection, neuroprotection, immune regulation, and chemoprevention of tumors [7, 8]. Consequently, resveratrol has attracted much attention as a research hotspot for the treatment of neurodegenerative diseases in recent years. A series of studies demonstrated that resveratrol had anti-AD effects by interfering with the formation of A*β*. Resveratrol could remodel the A*β* conformation selectively, transforming it into nontoxic multimers to reduce the neurotoxicity of A*β* [9]. Besides, resveratrol improved the spatial localization and memory of C67BL/6J mice [10], decreased the number of activated microglial cells in APP/Ps1 mice, and reduced inflammation caused by A*β* [11]. Since resveratrol has achieved acceptable results as a prominent natural compound for the treatment and prevention of neurodegenerative diseases, it was selected as a positive control group of experiments in our study.

Prevalence studies suggested that epigenetic modification of neurons should be accompanied by changes in the aging brain [12]; as an abnormal brain aging process, the epigenetic modification of AD was bound to change abnormally [13]. A series of studies have reported that the unbalance between 5-methylcytosine (5mC) and 5hmC is responsible for the disorder of DNA methylation; this was closely related to Alzheimer's disease, Parkinson's disease, and amyotrophic lateral sclerosis. Previous research illustrated that the levels of *TET1*, 5mC, and 5hmC increased significantly in preclinical AD and late AD patients while the levels of 5-formylcytosine and 5-carboxylcytosine decreased significantly [14]. Moreover, the levels of 5mC and 5hmC were remarkably increased in different brain areas of AD patients, accompanied by an increase in AD pathological molecular markers such as A*β* and Tau proteins [15]. However, other studies indicated the 5hmC levels in the entorhinal cortex and cerebellum regions of AD patients were noticeably reduced compared with normal individuals [16]. Furthermore, the apparent modifications of DNA in the hippocampus of the brains of 10 AD patients and normal subjects were compared [17]. The results demonstrated that the contents of 5mC and 5hmC in the hippocampus of AD patients decreased by 19.6% and 20.1%, respectively, and were negatively correlated with the level of amyloid plaques in the hippocampus. Although different studies on 5hmC levels in the brain of AD patients had opposite conclusions, the dysfunction of 5hmC and TETs proteins played an essential role in the occurrence and development of AD diseases; meanwhile, the mechanism of action needs to be further investigated.

## 2. Materials and Methods

### 2.1. Material

The human cell line SHSY-5Y was purchased from Kunming Cell Bank of the Chinese Academy of Sciences. Cordycepin was purchased from Shanghai Shifeng Biological technology CO., LTD. Cordycepic acid was prepared by ethanol extraction (purity ≥ 95%). Resveratrol was purchased from Sigma. The wild type (WT) and transgenic (App/Ps1/Tau, AD) mice used in the experiment were all C57BL/6J mice.

### 2.2. Cell Culture

As being derived from humans, SHSY-5Y cells are often used to mimic responses of neurons in vitro models. They can be induced by all-trans-retinoic acid (RA) and obtain some neuron-like properties. The procedure of culturing SHSY-5Y cells were performed as described with modification (Cell culture protocol for SHSY-5Y neuroblastoma cells (human, ATCC# CRL-2266)). Briefly, SHSY-5Y cells were cultured with a mixture of complete medium (DMEM, high glucose), 10% heat-inactivated fetal bovine serum (FBS), penicillin (50 U/mL), streptomycin (50 mg/mL), and incubated in a humidified, 5% CO_2_, 37°C incubator.

For IF, cells at 6 × 10^3^ − 8 × 10^3^ per well were cultured into 24-well plate (with slides). For Dot Blot and qPCR, cells at 2 − 3 × 10^4^ per well were cultured into 6-well plates. 24 h after seeding, change the medium to induced medium (neurobasal, 2% B27, 1% glutamine, penicillin (50 U/mL), streptomycin (50 mg/mL), and 0.1% RA), and add the different compounds with different concentration at the same time. Change the induced medium every other day, fixed cells after 48 h or 96 h.

### 2.3. Immunofluorescence

The procedure of immunofluorescence (IF) was performed as described with modification (Abcam Technical Manual (2015)). Briefly, after fixed cells, using rabbit antibody to Map2 or rH2ax as the primary antibody, incubated overnight at 4°C. Horseradish peroxidase-conjugated antibody to rabbit (Sigma), was used as secondary antibody, and incubated for 1 h at room temperature.

### 2.4. RNA Preparation and qPCR

RNA was isolated from SHSY-5Y cells with RNeasy mini Kit (Qiagen), stored at -80°C after purity test and concentration test by NanoDrop2000.

### 2.5. Genomic DNA Preparation and Dot Blot

Genomic DNA was isolated from SHSY-5Y cells with PureLink TM Genomic DNA Purification kits (Invitrogen), stored at -20°C after the purity test and concentration test by NanoDrop2000. Dot blots were performed on a Bio-Dot Apparatus as described previously [18]. Using the rabbit antibody to 5hmC (#39769, Active Motif) as the primary antibody, incubated overnight at 4°C. Horseradish peroxidase-conjugated antibody to rabbit (Sigma) was used as a secondary antibody, and incubated for 30 min at 20–25°C. Standard DNA templates were loaded for the quantification and to verify the specificity of antibodies.

### 2.6. Isolation of Primary Neuron

The cerebral cortex was removed from 16.5-day wild type (WT) and mutant fetal mice. 0.5 mm thickness slices were made perpendicular to the long axes of the cortex and transferred to a tube at 4°C with B27 (Cat. No. 17504) and 0.5 mM L-glutamine (Cat. No. 25030). Slices were digested at 37°C for 10 min with 0.25% trypsin, added medium with 10% FBS to stop the reaction. After triturated 10 times with a siliconized 9-inch Pasteur pipette, the pieces were cultured in a 6-well plate with B27 (Cat. No. 17504) and 0.5 mM L-glutamine (Cat. No. 25030).

### 2.7. Statistical Analysis

Data were expressed as mean ± variance of mean of at least three independent experiments. All values were analyzed using one-way ANOVA. *p* values < 0.05 were considered significant.

## 3. Results and Discussion

According to our previous studies, cordycepin increased cells' number at 20 *μ*M while it inhibited the proliferation of SHSY-5Y at higher concentrations (80 *μ*M), indicating a significant reduction in cells' number. The toxicity of cordycepic acid on SHSY-5Y cells was not obvious; the cells' number did not exhibit a significant decrease even after the treatment at 220 *μ*M. In this study, resveratrol was selected as a positive control group, the highest concentration at 10 *μ*M.

### 3.1. DNA Damage of SHSY-5Y Cells after Different Treatment

**Figure 1:**
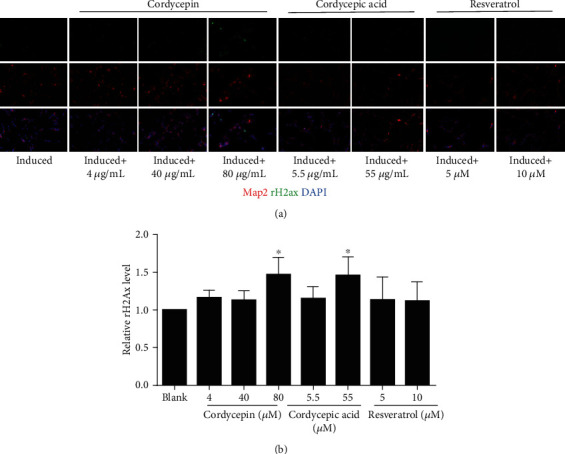
Effect of DNA stability after different treatments on SHSY-5Y induced neuron. (a) Immunofluorescence diagram. (b) Expression quantity of rH2ax. (^∗^*p* < 0.05 vs. blank).

The generation and accumulation of DNA damage are one of the crucial causes of neuronal death in neurodegenerative diseases. Therefore, the cells were treated with cordycepin, cordycepic acid, and resveratrol for 96 h. Then, rH2Ax fluorescence staining was performed to detect DNA damage (Figure 1(a)).

Under the treatment of low-dose cordycepin (20 *μ*M, 40 *μ*M), there was no significant DNA damage while the level of DNA damage marker rH2Ax was remarkably increased by cordycepin at a high dose (80 *μ*M). Besides, cordycepic acid also exhibited no significant DNA damage at low dose (5.5 *μ*M) treatment and displayed significant DNA damage at a treatment concentration of 55 *μ*M. Moreover, resveratrol presented no significant DNA damage at both selected concentrations (5 *μ*M and 10 *μ*M) (Figure 1(b)).

### 3.2. Expression of 5hmC after Different Treatment in SHSY-5Y

**Figure 2:**
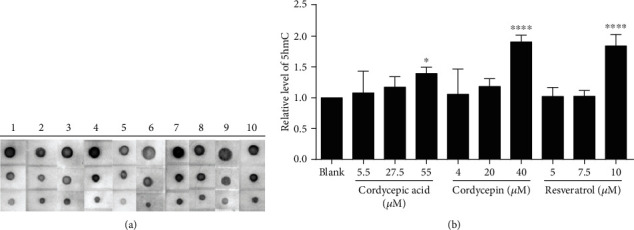
Result of Dot Blot. (a) Different treatment: (1) blank, (2) cordycepic acid 5.5 *μ*M, (3) cordycepic acid 27.5 *μ*M, (4) cordycepic acid 55 *μ*M, (5) cordycepin 4 *μ*M, (6) cordycepin 20 *μ*M, (7) cordycepin 40 *μ*M, (8) resveratrol 5 *μ*M, (9) resveratrol 7.5 *μ*M, (10) resveratrol 10 *μ*M. (b) Expression quantity of 5hmC after different treatments. (^∗^*p* < 0.05 vs. blank, ^∗∗∗∗^*p* < 0.0001 vs. blank).

5hmC is an intermediate product of DNA demethylation and directly involved in DNA demethylation. In this study, the SHSY-5Y cells were treated with different compounds for 4 days, and the level of 5hmC was detected through a Dot Blot assay (Figure 2(a)) to verify the effect of different compounds on the expression of 5hmC in neurons.

5hmC, as an independent and stable modified base, plays an essential role in the regulation of gene expression. The content of 5hmC was higher in stem cells and central nervous cells, suggesting its importance in regulating brain development [19, 20]. Previous studies revealed that 5hmC was positively correlated with abundant gene expression in mouse cerebral cortex and cerebellar nerve cells, indicating that 5hmC might promote gene transcription and expression [21]. Our result illustrated that the expression level of 5hmC increased noticeably at cordycepic acid 55 *μ*M (*p* < 0.05), cordycepin 40 *μ*M (*p* < 0.0001), and resveratrol 10 *μ*M (*p* < 0.0001); besides, the expression level of 5hmC was generally proportional to the treatment concentration (Figure 2(b)). It was verified that the enrichment of 5hmC in SHSY-5Y cells was promoted by high concentrations of cordycepic acid, cordycepin, and resveratrol.

Combined with the above experimental results, lower but harmless concentrations (cordycepin 20 *μ*M, cordycepic acid 27.5 *μ*M, and resveratrol 7.5 *μ*M) were selected for the subsequent experiments.

### 3.3. Detection of DNA Methylation-Related Gene

Therefore, what exactly caused the different expressions of 5hmC after different treatments? Several genes related to DNA methylation and demethylation and some genes related to the growth of nerve cells were designed to obtain the corresponding conclusions.

The qPCR results indicated the expression levels of *TET1*, *TET2*, and *TET3*. Thymine DNA glycosylase (*TDG*) related to DNA demethylation had changed to varying degrees after the treatment with different compounds. The expression levels of *TET1* and *TET2* in the cordycepin group increased dramatically compared with the resveratrol group. The expression level of *TET3* was also increased noticeably compared with the blank group. The mRNA level of *TDG* gene increased reasonably in the cordycepin group and resveratrol group. It was suggested that cordycepin should have a better demethylation effect than cordycepic acid at a treatment concentration of 27.5 *μ*M.

Results of the DNA methyltransferase (DNMT) gene family test demonstrated that resveratrol significantly increased the expression of *DNMT3a*, *DNMT3b*, and *DNMT1*. However, the expression of DNMTs in cordycepic acid and cordycepin treated groups was significantly lower than that in resveratrol. Particularly, the expression of *DNMT3b* in the cordycepin group was significantly lower compared to the blank group. These results suggest that resveratrol might play a role in promoting the process of DNA methylation.

**Figure 3:**
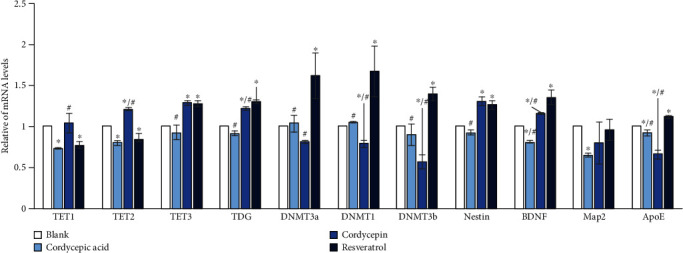
Result of qPCR (^∗^*p* < 0.05 vs. blank. ^#^*p* < 0.05 vs. resveratrol).

It can be observed from Figure 3 that the mRNA levels of *Nestin* increased remarkably after treated by cordycepin and resveratrol compared with the blank group; meanwhile, the *Nestin* expression level of cordycepic acid was significantly lower than that of resveratrol. It was indicated that the treatment of cordycepin and resveratrol should increase the number of neural precursor cells and might have a positive effect on subsequent neurogenesis. Simultaneously, the addition of different compounds had no effect on the expression of Sry-related HMG box and glial fibrillary acidic protein (GFAP) (not shown in the figure); none of them could promote the differentiation of nerve cells or form astrocytes.

A series of studies indicated that *ApoE* was related to pathological features of AD, and the formation of NFTs was driven by the interaction between *ApoE* and Tau protein [22]. Other studies suggested that *ApoE* exists in various forms of A*β*, promoting the formation of A*β* precipitation [23], or as a carrier or molecular chaperone for A*β* [24]. It was worth noting that the mRNA level of the *ApoE* gene in the cordycepin group was significantly decreased compared with the blank group and resveratrol group (*p* < 0.05). Therefore, cordycepin might hinder the formation of NFTs and the accumulation of A*β* by reducing the expression of *ApoE* in the brain; besides, it might affect the progression of AD. *ApoE* may enhance the scavenging of brain A*β* through promoting the effect of astrocytes to find, internalize, and degrade deposits of A*β* [25]. In the AD model, astrocytes are crucial mediators for A*β* to increase downstream neurotoxic events [26]. Nevertheless, our results demonstrated that cordycepin had no effect on the expression of GFAP, indicating that cordycepin may not act through modulation of astrocytic activity.

### 3.4. Effects of Different Drug Treatments on Mouse Primary Neurons

**Figure 4:**
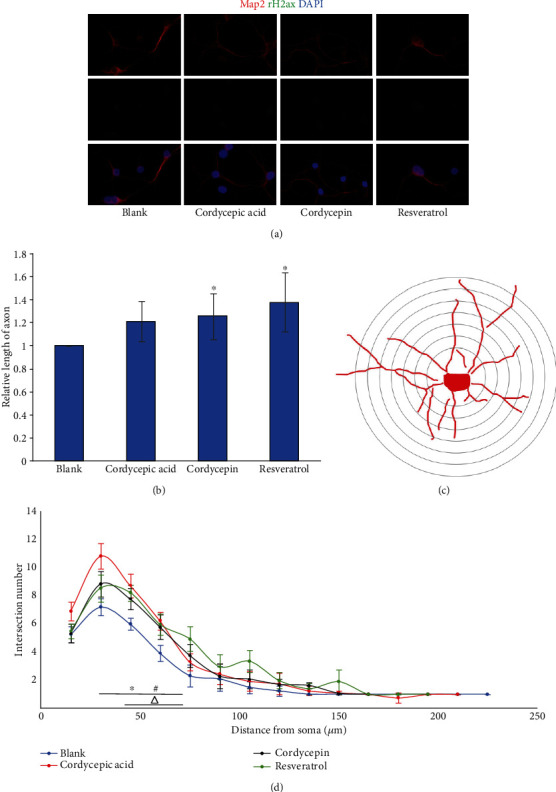
Effect of different treatments on primary neurons in WT mice. (a) Immunofluorescence staining of primary neurons in WT mice. (b) Change of axonal length after different treatment of WT primary neurons. (c) Sholl analysis diagram. (d) The dendritic branching of primary neurons in WT mice treated with different compounds. (^∗^*p* < 0.05, cordycepic acid vs. blank; ^#^*p* < 0.05, cordycepin vs. blank; ^△^*p* < 0.05, resveratrol vs. blank).

The primary cortical neurons of WT mice were used for culture. Different compounds were added 72 hours later. Afterward, the effects of different compounds on the length of axons and dendrites of neurons were observed. After the treatment of primary neurons by adding 20 *μ*M cordycepin and 7.5 *μ*M resveratrol, the length of axons was increased significantly compared to the blank group (*p* < 0.05), and there was no significant difference compared with the blank group (*p* > 0.05) after adding 27.5 *μ*M cordycepic acid, as illustrated in Figure 4(b). Regarding the dendritic branching of neurons (Figure 4(d)), the tendency of the three compound-adding groups and the blank group was generally the same, peaking at 30 *μ*m. It was indicated that the length of the dendrites of the neurons in each treatment group was approximately 30 *μ*m. Cordycepic acid and cordycepin reasonably increased the number of dendritic branches at the range of 30-75 *μ*m (*p* < 0.05), and cordycepic acid promoted more dendritic branches compared to cordycepin. Meanwhile, the number of dendritic branches after the treatment of resveratrol was different from the blank group at the range of 45-75 *μ*m. The immunofluorescence staining of primary neurons in WT mice (Figure 4(a)) illustrated that the morphology of neurons after microtubule-associated protein-2 (MAP2) staining was normal, with the clear structure, and there was no obvious effect on the DNA damage of primary neurons at the above three drug concentrations.

**Figure 5:**
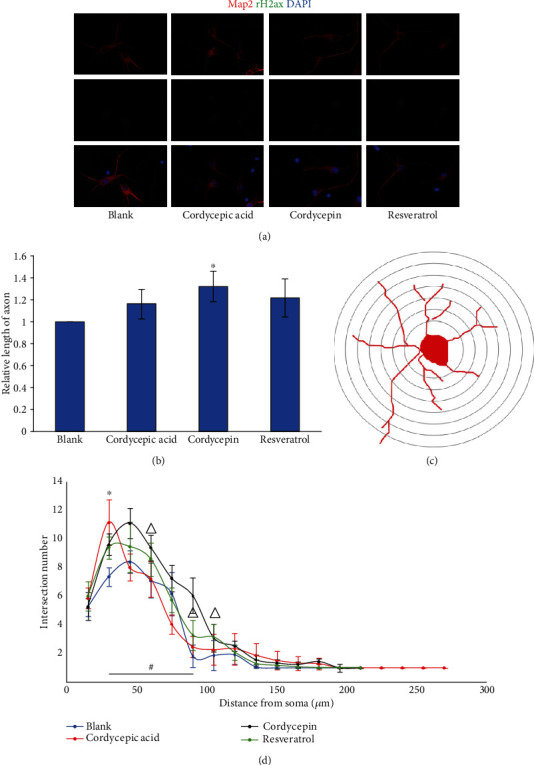
Effect of different treatments on primary neurons in AD mice. (a) Immunofluorescence staining of primary neurons in AD mice. (b) Change of axonal length after different treatment of AD primary neurons. (c) Sholl analysis diagram. (d) The dendritic branching of primary neurons in AD mice treated with different compounds. (^∗^*p* < 0.05, cordycepic acid vs. blank; ^#^*p* < 0.05, cordycepin vs. blank; ^△^*p* < 0.05, resveratrol vs. blank).

IF staining (Figure 5(a)) suggested that DNA damage of neurons was not visible under different treatment conditions, and the morphology of neurons after *MAP2* staining was intact. Besides, cordycepic acid and resveratrol exhibited no significant effect on the axon length of primary neurons in AD mice while cordycepin significantly increased the axon length of primary neurons in AD mice (Figure 5(b)). As demonstrated in Figure 5(d), the density of dendrites after treatment with cordycepic acid increased noticeably at 30 *μ*m, and there was no significant effect on the remaining length range. After the treatment of resveratrol, the dendritic branching tendency was close to that in the blank group, only increasing significantly at 60, 90, and 105 *μ*m. Noticeably, cordycepin remarkably increased the number of dendrites at 30-95 *μ*m, and the branching tendency was similar to that in the blank group, reaching the peak of branch number at 45 *μ*m. It was indicated that the dendritic branching of AD primary neurons was promoted by a suitable concentration of cordycepin.

## 4. Conclusions

Alzheimer's disease (AD), as a neurodegenerative disease, was characterized by selective neuron loss and degeneration in the hippocampus and cortex, associated with amyloid plaques and NFTs in the brain [1]. Besides, the incidence of AD was related to age. Specifically, the proportion of AD patients increased with the increase of age [27]. Therefore, finding compounds that can relieve AD symptoms has been a hot spot in recent years. Since previous studies revealed that methylation disorders occurred in the pathological brain regions of AD, our research focused on the discovery of natural compounds that specifically regulated 5hmC.

Cordycepic acid and cordycepin were selected as experiment materials, with resveratrol as the positive control group. It was observed from our experiments that cordycepic acid (55 *μ*M), cordycepin (40 *μ*M), and resveratrol (10 *μ*M) promoted the level of 5hmC. Researchers demonstrated that high-level 5hmC played an essential role in sustaining the function and survival of neurons [28]. Therefore, the accumulation of 5hmC might have a positive effect on neurons. However, cordycepic acid at 55 *μ*M and cordycepin at 40 *μ*M caused DNA damage compared with the blank group.

It was also discovered that cordycepic acid and cordycepin exhibited significant cytotoxicity at high concentrations. Meanwhile, cordycepin increased the expression quantity of TETs protein and reduced the DNMTs, indicating that cordycepin could promote the level of 5hmC by high expression of TETs. Nevertheless, cordycepic acid had no significant effect on the expression of methylation and demethylation-related genes. Particularly, the level of *ApoE* reduced significantly after treated by cordycepin; this result was related to the formation of NFTs and the accumulation of A*β* [23]. Recent studies illustrated that after changing the neuron structure of human *ApoE4*, the signs of AD were eliminated, and cell function and viability were improved [29]. In AD patients with *ApoE4*, the higher the tau level in cerebrospinal fluid (CSF), the worse the plasticity of long-term potentiation- (LTP-) like cortical, and the faster the disease progression [30]. The reduction of *ApoE* suggested that cordycepin might affect the formation of NFTs and A*β*, reduce the tau level in CSF, and restore the plasticity of LTP-like cortical. Moreover, the low expression of the *ApoE* level might reduce the risk of AD. Simultaneously, cordycepin promoted the axon length of primary neurons on both WT and AD mice and increased the dendritic branching. In conclusion, cordycepin might affect the progression of AD by regulating the level of 5hmC and improving the morphology of neurons.

No natural or chemical compounds that have been reported can regulate 5mC/5hmC to interfere with nervous system function or to improve the symptoms of neurodegenerative diseases at present. Therefore, this study might provide a new idea for research on neurodegenerative diseases. Besides, we attempt to improve the development of natural medicines for the prevention and treatment of neurodegenerative diseases and update the neuroprotective strategies through the mechanism study of cordycepin. However, this study only involved cellular levels; the specific effects of natural compounds (such as cordycepin) on AD or other neurodegenerative diseases have not been validated in animal experiments. The mechanism of action remains to be further investigated.

## Data Availability

The data used to support the findings of this study are included within the article.
